# Prioritizing Susceptible Genes for Thyroid Cancer Based on Gene Interaction Network

**DOI:** 10.3389/fcell.2021.740267

**Published:** 2021-08-23

**Authors:** Lin-kun Zhong, Chang-lian Xie, Shan Jiang, Xing-yan Deng, Xiao-xiong Gan, Jian-hua Feng, Wen-song Cai, Chi-zhuai Liu, Fei Shen, Jian-hang Miao, Bo Xu

**Affiliations:** ^1^Department of General Surgery, Zhongshan City People’s Hospital, Zhongshan, China; ^2^Intensive Care Unit, Zhongshan Hospital of Traditional Chinese Medicine Affiliated to Guangzhou University of Chinese Medicine, Zhongshan, China; ^3^Reproductive Medicine Center, Boai Hospital of Zhongshan, Zhongshan, China; ^4^Department of Thyrovascular Surgery, Maoming People’s Hospital, Maoming, China; ^5^Department of Thyroid Surgery, Guangzhou First People’s Hospital, School of Medicine, South China University of Technology, Guangzhou, China

**Keywords:** thyroid cancer, gene interaction, genetic testing, Adaboost, deep neural network

## Abstract

Thyroid cancer ranks second in the incidence rate of endocrine malignant cancer. Thyroid cancer is usually asymptomatic at the initial stage, which makes patients easily miss the early treatment time. Combining genetic testing with imaging can greatly improve the diagnostic efficiency of thyroid cancer. Researchers have discovered many genes related to thyroid cancer. However, the effects of these genes on thyroid cancer are different. We hypothesize that there is a stronger interaction between the core genes that cause thyroid cancer. Based on this hypothesis, we constructed an interaction network of thyroid cancer-related genes. We traversed the network through random walks, and sorted thyroid cancer-related genes through ADNN which is fusion of Adaboost and deep neural network (DNN). In addition, we discovered more thyroid cancer-related genes by ADNN. In order to verify the accuracy of ADNN, we conducted a fivefold cross-validation. ADNN achieved AUC of 0.85 and AUPR of 0.81, which are more accurate than other methods.

## Introduction

Thyroid carcinoma (TC) is the most common malignant tumor of endocrine system, accounting for 2.5% of all human cancers, accounting for 90% (Maniakas et al., 2020; Sahu and Pattanayak, 2020; Xia et al., 2020) are cell-derived thyroid malignancies are derived from the follicular cells, including follicular thyroid carcinoma (FTC), papillary thyroid carcinoma (PTC), poorly differentiated thyroid carcinoma (PDTC), and anaplastic thyroid carcinoma (ATC) (Dralle et al., 2015). PTC and FTC with low malignancy are classified as differentiated thyroid carcinoma (DTC), accounting for about 90% of all thyroid cancers (Zanella et al., 2021). And the majority of deaths from thyroid carcinoma was caused by ATC (Molinaro et al., 2017). Medullary thyroid carcinoma (MTC) originates from parafollicular (c) cells, accounts for 2–4% of all thyroid carcinoma (Ceolin et al., 2019; Chen et al., 2020). About 25% of MTC cases are caused by germline genetic mutations, that is, familial medullary thyroid carcinoma (FMTC), while 75% are sporadic cases. Hereditary cases can occur alone, it can also be used as a part of multiple endocrine neoplasia type 2 (men2) syndrome (Vijayan et al., 2021).

According to statistics, the incidence rate of thyroid malignancies in recent decades is almost entirely due to the improvement of diagnostic accuracy and over diagnosis of PTC tumors, while the incidence rate of FTC, ATC, and MTC remains relatively stable (Xing, 2019; Zhang et al., 2021). The degree of differentiation of PTC is relatively high, and the corresponding degree of malignancy is relatively low, but it is not equivalent to the low risk of PTC. There are generally no obvious symptoms in the early stage of PTC (Tsukatani et al., 2020), but once clinical symptoms appear, such as hoarseness, Tracheal compression, etc., usually have entered the local advanced stage, and the best time for treatment has been missed at this time, and the metastasis of cervical lymph nodes and the invasion of local muscles, nerves and other tissues can often be seen during surgical treatment, resulting in postoperative complications. The treatment effect is not satisfactory.

Molecular markers are an effective tool for diagnosis, especially for thyroid nodules whose Fine needle aspiration cytology (FNAC) is uncertain (Sanguedolce et al., 2015). Gene mutation and chromosome rearrangement are important genetic changes in the occurrence and development of thyroid cancer. The molecular pathogenesis of most thyroid cancer involves mitogen activated protein kinase (MAPK) and phosphatidylinositide 3-kinases/protein kinase B (Sui et al., 2014), PI3K/Akt signaling pathway (Petrulea et al., 2015) is out of balance. BRAF and RAS point mutations, RET/PCT and Pax8/PPAR γ Rearrangement can activate MAPK pathway, and mainly occurs in DTC. BRAF mutation and RET/PCT rearrangement are common in PTC, while Ras mutation and Pax8/PPAR γ Rearrangement is a common molecular change in FTC. Pik3/Akt pathway is mainly activated by Ras, TP53 and TERTP mutations. TP53 and TERTP mutations are rare in well-differentiated thyroid cancer (Penna et al., 2016), and the mutation frequency is high in ATC and PDTC, which may be related to tumor invasion. Therefore, FNAC is difficult to determine the benign and malignant thyroid nodules, which can be combined with relevant molecular detection to help diagnosis, so as to improve the diagnostic accuracy of thyroid cancer.

According to the data released by the Cancer Genome Atlas (TCGA) in 2014 (Tomczak et al., 2015), 402 patients with thyroid cancer were analyzed. Compared with other cancers, the frequency of gene somatic mutations in thyroid cancer is relatively low. The frequency of BRAF V600 was 58.5%, which was the highest mutation site in thyroid cancer. In addition, the high-frequency mutation gene also includes three RAS gene family members, such as NRAS and KRAS, which are known tumor related genes, with a mutation frequency of 12.9% in European and American populations. In addition, some new thyroid cancer driving genes eif1ax, ppm1d, and CHEK2 were identified, and some of them also had gene fusion (Agrawal et al., 2014). TCGA research, with large sample size and various analysis methods, not only found a large number of somatic mutations, but also copy number variation and gene fusion information, which has great reference value and clinical significance. BRAF gene is used to assist in the diagnosis of benign and malignant thyroid nodules (Salvatore et al., 2006), which greatly reduces the misdiagnosis rate in clinical diagnosis and improves the accuracy of preoperative diagnosis of patients with papillary thyroid cancer. BRAF gene is used for clinical diagnosis of papillary thyroid cancer (Trovisco et al., 2004). It can also be used as an important factor for clinical prediction of post-operative recurrence and guidance of medication, so as to facilitate the formulation of individualized and precise diagnosis, treatment and follow-up plans. In recent years, deep learning methods have been widely used in the diagnosis thyroid cancer. Lee JH et al. developed a deep learning-based computer-aided diagnosis (CAD) system could accurately classify cervical lymph node metastasis (LNM) on CT images in patients with thyroid cancer (Lee et al., 2019). Li X et al. indicated that compared with a group of skilled radiologists, deep convolutional neural network (DCNN) models that showed similar sensitivity and improved the diagnostic accuracy of thyroid cancer on sonographic images (Li et al., 2019).

Although multiple TC-related genes have been found by collecting samples and implementing gene differential expression analysis (Zhao et al., 2021b), people are still unclear about the pathogenesis and early diagnosis of thyroid cancer. With the increasing computational power and omics data, machine learning methods can identify disease-related molecules on a large scale to reveal the pathogenesis (Zhao et al., 2020c), disease occurrence process (Zhao et al., 2020a) and clinical medication guidance (Tianyi et al., 2020). Most of the calculation methods are based on similarity and interaction (Zhao et al., 2020b, 2021a). In this article, we propose hypotheses: there is a stronger interaction between the core genes that cause thyroid cancer. There is a close relationship between the pathogenic genes of thyroid cancer, but the interaction between the genes only related to thyroid cancer and these genes is not that close. Based on this hypotheses, we constructed a gene interaction network and used Random Walk (RW) to traverse this network. Then, Adaboost and deep neural network (DNN) was fused to identify TC-related genes.

## Materials and Methods

**FIGURE 1:**
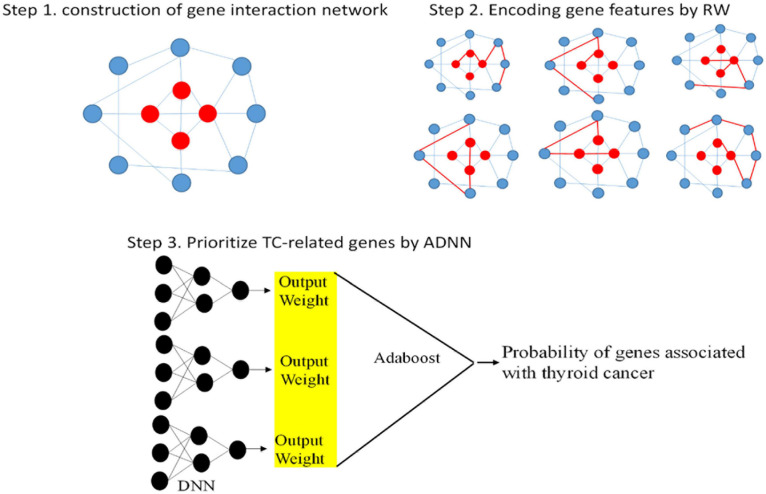
Three steps of ADNN to prioritize genes related to thyroid cancer.

There are three steps to implement ADNN. First, TC-related genes are obtained from DisGeNET (Piñero et al., 2015). Then, we collected genes which can interaction with TC-related genes to construct gene interaction network. The red points represent TC-related genes and blue points represent other genes. The second step is to use RW to traverse this network. The features of genes can be encoded by this step. The last step is to fuse Adaboost with DNN to prioritize TC-related genes. The whole process of ADNN is shown as Figure 1.

### Construction of Gene Interaction Network

**FIGURE 2:**
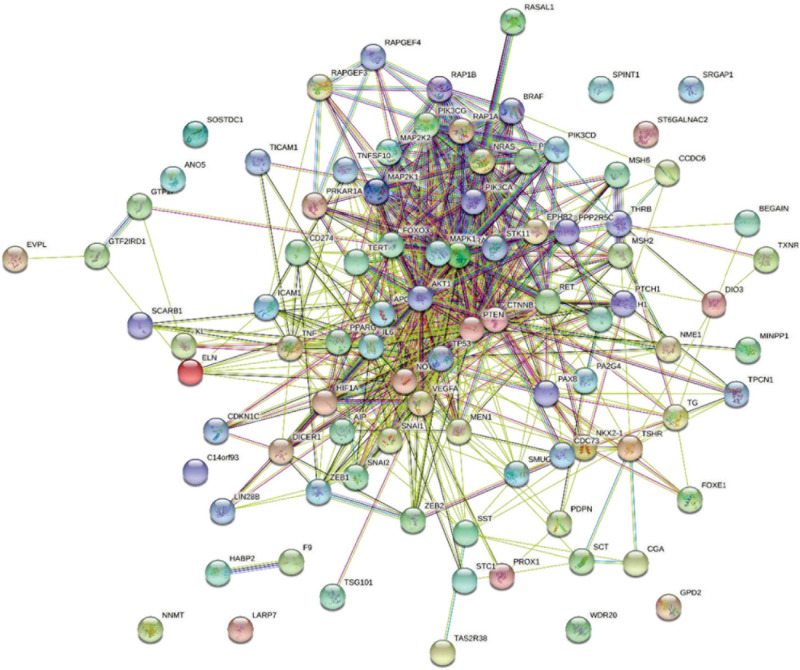
Gene interaction network of TC-related genes.

First, we obtained TC-related genes from DisGeNET. According to DisGeNET, there are 147 genes related to TC. Using String database (Szklarczyk et al., 2016), we draw these genes interaction network as Figure 2.

As we can see in Figure 2, the interaction between some core genes in the center of the network is very close. Although there are still some marginal genes that cannot interact with other genes, most of the genes have close relationship with other genes. We collected genes which can interaction with 147 TC-related genes to construct a whole gene interaction network.

### Encoding Gene Features by RW

Since the gene interaction network we construct is a two-dimensional graph, when we walk through the network in a probabilistic manner based on gene interaction, when the node information of the current gene is known, the historical gene node traversal information and the future gene node The traversal path is irrelevant. Therefore, we can regard the genetic coding method based on random walk as a Markov chain. In each step of the Markov chain, the probability distribution of gene interaction can change from one state to another, or maintain the current state. The change of state is called transition, and the probability associated with different states is called transition probability.

If A is the adjacency matrix of the gene interaction network, we can normalize A as:

(1) $$P=D^{-1}\,A$$

D is a diagonal matrix and the degree matrix of the gene interaction network:

(2) D⁢(i,i)=∑A⁢(i,j)

P is a random walk matrix, the transition probability of each node is 1, and P is the probability matrix associated with TC and all genes.

A random walk matrix corresponds to a Markov chain, and the probability distribution of TC-related genes changes as the state in the Markov chain changes. Starting from any state, the probability of going to the next state is as follows:

(3) $$P_{t+1}=A_{t}\,P$$

This process continues, and the relationship between TC and genes is constantly changing. After a period of time, it reaches a state of equilibrium. The equilibrium state is also called steady state, which means that the probability distribution of the association between TC and genes no longer changes. The calculation method of steady state is as follows:

(4) $$\pi =D\,\left(i,j\right)/\sum_{i}\sum_{j}A\,\left(i,j\right)$$

When π*P* = π, the entire system reaches a steady state. This steady state is the final calculated association between TC and gene.

### Prioritize TC-Related Genes by ADNN

DNN neural network layers can also be simply divided into three categories: input layer, hidden layer and output layer. Its layers are fully connected, that is, all neurons in the upper layer are connected to any neuron in the next layer. Its partial model is:

(5) O=σ⁢(∑w⁢x+b)

*O* is output. σ() is activation function. *w* is the coefficient of linear relationship, *b* is bias model parameters.

Using DNN network architecture to identify the interaction pattern between TC and gene, we need to define the objective function to measure the loss of model fitting.

(6) $$J\,\left(w,b,x,y\right)=\frac{1}{2}\,{∥a^{L}-y∥}_{2}^{2}$$

The process of training DNN is to minimize the loss function. The parameters of DNN model is shown in Table 1.

**TABLE 1:** The parameters of DNN model.

**Structure**	**Parameters**
Layer 1	Units: 256 Activation function: Tanh Dropout rate: 0.3
Layer 2	Units: 128 Activation function: Tanh Dropout rate: 0.2
Layer 3	Units: 2 Activation function: Tanh
Loss function	Binary cross entropy
Optimizer	RMSprop

Due to the small sample set, DNN is used as a weak classifier. In order to make the model more accurate, we introduced AdaBoost.

First, set the initial weight of each sample to 1/N. Then, training samples to get the first DNN model, test this DNN model, increase the weight of the unclassified correct samples and reduce the weight of the classified correct samples. At the same time, the weight of the DNN model is obtained. Repeating the above process, we can get multiple DNN models and corresponding weights, thereby obtaining the final strong classifier.

The error rate of each model can be calculated as following:

(7) $$err_{m}=\sum_{i=1}^{N}w_{m\,i}||\left(G_{m}\left(x_{i}\right)\neq y_{i}\right)$$

The weight of the model is:

(8) $$a_{m}=\frac{1}{2}\,\log\frac{1-e\,r\,r_{m}}{e\,r\,r_{m}}$$

The final model is the summary of all DNN models:

(9) $$G\,\left(x\right)=\arg\max\,\sum_{m:G_{m}\,\left(x\right)=y}a_{m}$$

## Results

**FIGURE 3:**
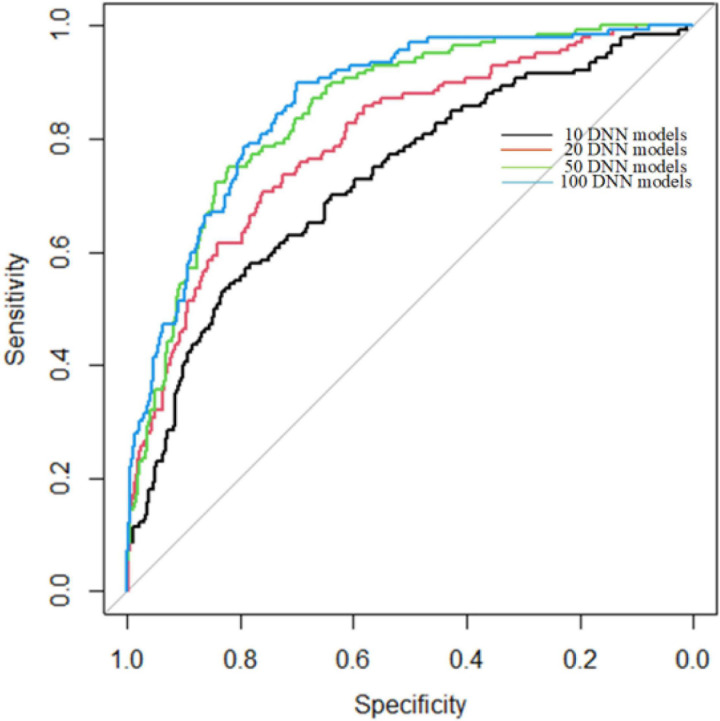
ROC curves of different numbers of DNN models.

Since we used DNN as a weak classifier and the number of DNN models is set by experience, we used 5-cross validation to find the best number of DNN models of ADNN. The process of 5-cross validation is to divide whole sample set into five groups. We used one group for testing and four groups for training each time. After repeating five times, each group has been tested once and trained four times. We use 10, 20, 50, and 100 DNN models to build ADNN, respectively. The experiment results are shown as Figure 3.

In Figure 3, the AUC are 0.73, 0.79, 0.85, 0.86 for 10, 20, 50, 100 DNN models, respectively. As shown in Figure 3, the AUC of 50 and 100 DNN models are very close. However, constructing 100 DNN models is twice time consuming than 50 DNN models. Therefore, we used 50 DNN models to build ADNN model.

In order to show the superiority of ADNN, we compared it with several other methods such as DNN, ASVM, ANB, Random Forest (RF). ASVM is the fusion of Adaboost and Support Vector Machine (SVM). ANB is the fusion of Adaboost and Naïve Bayes (NB).

The comparison results is listed in Table 2.

**TABLE 2:** Comparison of ADNN and other methods.

**Method**	**AUC**	**AUPR**
ADNN	0.85	0.81
DNN	0.69	0.65
ASVM	0.82	0.79
ANB	0.76	0.71
RF	0.78	0.76

Compared ADNN with ASVM, we can find that DNN is more suitable than SVM in prioritizing susceptible genes for thyroid cancer. Compared ADNN with DNN, we can find that Adaboost can significantly increase the accuracy of prioritizing susceptible genes for thyroid cancer.

## Conclusion

Genetic factors are an important cause of thyroid cancer. Exploring the susceptibility genes of thyroid cancer is the key to understanding the pathogenesis and developing new treatment options. Collecting samples from patients and healthy individuals and analyzing differential gene expression is very costly and time-consuming. After years of research, researchers have found only 147 genes related to thyroid cancer. The role of these genes in thyroid cancer is unknown. In addition, there are more genes associated with thyroid cancer. To prioritize susceptible genes of thyroid cancer in large-scale, we proposed a novel method, named ADNN, to identify TC-related genes by gene interaction network. We constructed gene interaction network based on known TC-related genes and used RW to encode the features of genes. Then, we fused Adaboost with DNN to classify whether a gene is related to TC and obtain the probability of genes associated with TC. We get the best number of DNN models needed to construct ADNN through experiments. Finally, we compared ADNN with several other methods. Overall, we propose a precise and efficient method for prioritizing susceptible genes for thyroid cancer.

## Data Availability Statement

The datasets presented in this study can be found in online repositories. The names of the repository/repositories and accession number(s) can be found in the article/supplementary material.

## Ethics Statement

Ethical review and approval was not required for the study on human participants in accordance with the local legislation and institutional requirements. Written informed consent for participation was not required for this study in accordance with the national legislation and the institutional requirements.

## Author Contributions

L-KZ, C-LX, SJ, J-HM, and BX participated in the study design. L-KZ, C-LX, SJ, X-XG, J-HF, W-SC, C-ZL, and FS analyzed the data. L-KZ, C-LX, SJ, and X-YD wrote the manuscript. All authors read and approved the final manuscript.

## Conflict of Interest

The authors declare that the research was conducted in the absence of any commercial or financial relationships that could be construed as a potential conflict of interest.

## Publisher’s Note

All claims expressed in this article are solely those of the authors and do not necessarily represent those of their affiliated organizations, or those of the publisher, the editors and the reviewers. Any product that may be evaluated in this article, or claim that may be made by its manufacturer, is not guaranteed or endorsed by the publisher.
